# Ictal autonomic changes as a tool for seizure detection: a systematic review

**DOI:** 10.1007/s10286-018-0568-1

**Published:** 2018-10-30

**Authors:** Anouk van Westrhenen, Thomas De Cooman, Richard H. C. Lazeron, Sabine Van Huffel, Roland D. Thijs

**Affiliations:** 1 Stichting Epilepsie Instellingen Nederland (SEIN), Heemstede, P.O. Box 540, 2130 AM Hoofddorp, The Netherlands; 2 Department of Neurology, Leiden University Medical Center (LUMC), Leiden, The Netherlands; 3 Department of Electrical Engineering (ESAT), STADIUS Center for Dynamical Systems, Signal Processing and Data Analytics, KU Leuven, Louvain, Belgium; 4 IMEC, Louvain, Belgium; 5 Academic Center of Epileptology Kempenhaeghe, Heeze, The Netherlands; 6 Faculty of Electrical Engineering, Technical University Eindhoven, Eindhoven, The Netherlands

**Keywords:** Automatic seizure detection, Autonomic function(s), Autonomic parameter(s), Algorithm(s), Epilepsy, SUDEP

## Abstract

**Purpose:**

Adequate epileptic seizure detection may have the potential to minimize seizure-related complications and improve treatment evaluation. Autonomic changes often precede ictal electroencephalographic discharges and therefore provide a promising tool for timely seizure detection. We reviewed the literature for seizure detection algorithms using autonomic nervous system parameters.

**Methods:**

The PubMed and Embase databases were systematically searched for original human studies that validate an algorithm for automatic seizure detection based on autonomic function alterations. Studies on neonates only and pilot studies without performance data were excluded. Algorithm performance was compared for studies with a similar design (retrospective vs. prospective) reporting both sensitivity and false alarm rate (FAR). Quality assessment was performed using QUADAS-2 and recently reported quality standards on reporting seizure detection algorithms.

**Results:**

Twenty-one out of 638 studies were included in the analysis. Fifteen studies presented a single-modality algorithm based on heart rate variability (*n* = 10), heart rate (*n* = 4), or QRS morphology (*n* = 1), while six studies assessed multimodal algorithms using various combinations of HR, corrected QT interval, oxygen saturation, electrodermal activity, and accelerometry. Most studies had small sample sizes and a short follow-up period. Only two studies performed a prospective validation. A tendency for a lower FAR was found for retrospectively validated algorithms using multimodal autonomic parameters compared to those using single modalities (mean sensitivity per participant 71–100% vs. 64–96%, and mean FAR per participant 0.0–2.4/h vs. 0.7–5.4/h).

**Conclusions:**

The overall quality of studies on seizure detection using autonomic parameters is low. Unimodal autonomic algorithms cannot reach acceptable performance as false alarm rates are still too high. Larger prospective studies are needed to validate multimodal automatic seizure detection.

**Electronic supplementary material:**

The online version of this article (10.1007/s10286-018-0568-1) contains supplementary material, which is available to authorized users.

## Introduction

Epileptic seizures are potentially dangerous as they can lead to complications, including injury, status epilepticus, and sudden unexpected death in epilepsy (SUDEP) [1]. Adequate seizure detection may have the potential to minimize these complications and to ameliorate treatment evaluation, as seizures—particularly those at night—are often underreported [2–5]. Detection devices may also help to improve the independence and quality of life of people with epilepsy and their caregivers [3, 6].

Several parameters, including movement, sound, and autonomic nervous system changes, can be used to detect seizures. This review focuses on changes in autonomic function, including cardiovascular, respiratory, and transpiration changes [7]. Seizures can alter autonomic function, particularly if the central autonomic network is involved. The most common expression is a sudden increase in sympathetic tone [7, 8]. Ictal tachycardia (IT) is a very frequent sign, with prevalence rates ranging from 80 to 100% [9, 10]. IT is a hallmark of convulsive seizures (i.e., focal to bilateral tonic–clonic as well as generalized tonic–clonic seizures), and more common in temporal lobe vs. extratemporal lobe seizures [9]. Changes in autonomic function can precede ictal electroencephalographic (EEG) discharges by several seconds [10–12]. Preictal tachycardia has an incidence rate of approximately one-third of seizures [13]. Autonomic alterations may therefore provide an adequate tool for early seizure detection and facilitate timely interventions. Ictal arrhythmias and desaturations are more common but are thought to be self-limiting, while postictal arrhythmias and apneas may lead to SUDEP [14–17]. SUDEP usually occurs several minutes after a convulsive seizure (mean 10 min, range 2–17 min) [18]. Raising an alarm at seizure onset may be sufficient to allow timely intervention.

We aimed to systematically review different seizure detection algorithms based on autonomic function changes.

## Methods

This systematic review was conducted in accordance with the preferred reporting items for systematic reviews and meta-analyses (PRISMA) guideline [19].

**Fig. 1:**
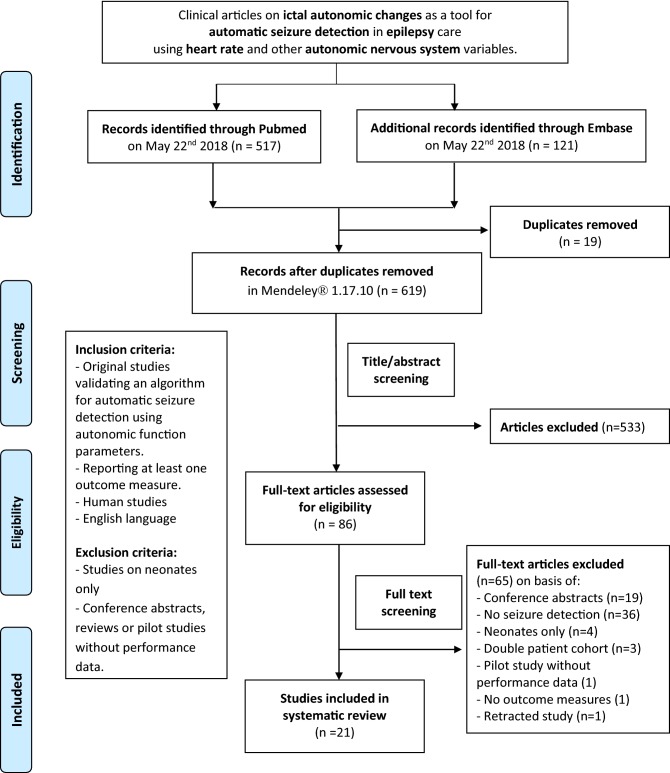
Flowchart of the search for applicable studies

The PubMed and Embase databases were systematically searched through May 2018 for original studies validating an algorithm for automatic seizure detection based on heart rate (HR), heart rate variability (HRV), oxygen saturation (SpO2), electrodermal activity (EDA, reflecting changes in transpiration), or a combination of the aforementioned. A sequence of synonyms for ‘autonomic variables,’ ‘seizures,’ and ‘detection’ were used as search terms (see Table S1 in the Electronic supplementary material, ESM). Studies were included if they met the following criteria: (1) human studies; (2) written in English; (3) reporting on children or adults with any type of epilepsy; (4) validating an algorithm for automatic seizure detection using autonomic parameters; (5) reporting at least one performance measure [sensitivity, positive predictive value (PPV), false alarm rate (FAR), or detection latency (DL)]. Studies on neonates only were excluded, because both seizure and autonomic function characteristics differ greatly at this age compared to older age. Pilot studies lacking performance data, as well as conference abstracts and reviews were also excluded (Fig. 1).

One author (AvW) screened all titles and abstracts, as well as the full texts of the remaining studies. For each article included, the following parameters were recorded: method of automatic seizure detection, type of autonomic variable, individual characteristics, number and types of seizures analyzed, prospective or retrospective validation, total recording time and performance of the algorithm (including sensitivity, PPV, FAR, and DL). We compared algorithm performance using multimodal autonomic parameters versus those using single modalities, provided that the studies (1) had a similar design (prospective vs. retrospective) and (2) reported both sensitivity and FAR.

The quality of the included studies was evaluated using the QUADAS-2 [20]. This tool consists of four domains (patient selection, index test, reference standard, and flow and timing) and different signaling questions to assist in judgments of the risk of bias and applicability. Additionally, we assessed all included studies according to the recently proposed standards for clinical validation of seizure detection devices (SDDs) [21].

## Results

Out of the 638 articles identified, 86 studies were selected on the basis of title and abstract. After full-text screening, 21 studies were included for further analysis. Most of the excluded articles lacked the validation of a seizure detection algorithm (see Fig. 1). The characteristics of the included studies are summarized in Table 1. Most of the studies (*n* = 15) focused on ictal cardiac changes as a tool for seizure detection algorithms, including HRV (*n* = 10) [8, 22–30], HR (*n* = 4) [31–34], and changes in QRS morphology (*n* = 1) [35]. Six studies used multimodal algorithms, including combinations of HR, corrected QT interval (QTc), SpO2, EDA, and accelerometry (ACC) [2, 36–40]. None of the included studies validated an algorithm based on oxygen saturation or EDA alone. Most studies were conducted in adults, but two studies included a pediatric population [23, 40], and six studies included both children and adults [22, 25, 35–37, 39]. Fourteen studies prospectively enrolled their participants [8, 22, 23, 26, 28, 30–33, 36–40], but only two studies prospectively validated their algorithm [31, 33].

**Table 1:** Characteristics of included studies

Study	Autonomic parameter	Measurement	Device	Algorithm	Prospective/retrospective validation	Population (*N*)	No. of seizures/TRT^a^	Type of seizures (*N*)	Mean age (years) [range]	Performance (mean of values per person)
De Cooman et al. [22]	Cardiac	HRV	Single-lead ECG	*Noise filtering*: High- and low-pass Butterworth filters. HRI-extract algorithm and HRI feature extraction with patient-independent SVM classifier: LOPO-CT. Fastened HRI-SVM seizure detection	Retrospective	Refractory temporal lobe epilepsy (17)	127/918 h	FOIA, FOBTC	33.5 [9–54]	Sens: 83.2% [50–100%] (overall: 81.9%) PPV: 7.9% [0.4–21%] (overall: 5.4%) FAR: 2.01/h [0.88–3.52/h] (overall: 1.97/h) DL: 13.3 s [− 18.2–54.3] (overall:17.8 s)
De Cooman et al. [23]	Cardiac	HRV	Single-lead ECG	See De Cooman [22]. Patient-specific heuristic adaptive classifier	Retrospective	1) Children (14) 2) Other group of children (14)	107/695 h	GOS (30), FOS (77)	NA	Patient-independent: Sens: (overall:81.3%) PPV: NA FAR (overall: 0.75/h) DL: NA Patient-specific: Sens: (overall: 77.6%) PPV: (overall: 30.7%) FAR (overall: 0.33/h) DL: 19.1 s
De Cooman et al. [24]	Cardiac	HRV	Single-lead ECG	See De Cooman [22]. Patient-specific heuristic adaptive classifier and real-time adaptive classifier	Retrospective	Temporal lobe epilepsy (19)	153/2833 h	FOS, FOIA, FOBTC, U, subclinical	NA	Patient-independent: Sens: (overall: 78.4%) PPV: (overall: 2.4%) FAR: (overall: 1.73/h) DL: NA Patient-specific: Sens: (overall: 76.5%) PPV: (overall: 3.7%) FAR: (overall: 1.09/h) DL: NA Adaptive: Sens: (overall: 77.1%) PPV: (overall: 3.3%) FAR: (overall: 1.24/h) DL: NA
Fujiwara et al. [25]	Cardiac	HRV	ECG	*Time domain analysis*: mean NN, SDNN, RMSSD, TP, NN50. * Frequency domain analysis*: LF, HF, LF/HF. Analysis over 2-5 min. Algorithm 2:* T*^2^ and* Q* statistics exceed limit > 10 s continuously	Retrospective	Refractory focal epilepsy (14)	11/69 h	FOS, awakening seizures (11)	30.6 [14–63]	T^2^ (Overall): Sens: 55% PPV: NA FAR: 1.2/h DL: − 524 ± 216 s Q (Overall): Sens: 91% PPV: NA FAR: 0.7/h DL: − 494 ± 262 s
Jeppesen et al. [26]	Cardiac	HRV	Single-lead ECG	*Noise filtering*: High-pass filter + manual edit. Automatic R-peak detection. *Lorenz plot*^b^* analysis*: SD1, SD2, CSI, mCSI, CVI	Retrospective	Temporal lobe epilepsy (5)	11/13 h	FOIA (11)	NA	Sens: 88% (CSI-30), (overall: 73%, CSI-30, mCSI-50) PPV: NA FAR: NA DL: − 5–60 s
Jeppesen et al. [8]	Cardiac	HRV	Single-lead ECG	*Noise filtering*: High-pass filter + manual edit. Automatic R-peak detection. * Frequency domain analysis*: HF-power (using FFT) HR-diff. *Lorenz plot*^b^* analysis*: CSI, mCSI	Retrospective	Focal epilepsy (17)	47/±27 h	FOS (44), FOBTC (3)	39 [20–55]	Sens: 81% (mCSI-100) (overall: 74%, mCSI-100) PPV: NA FAR: NA DL: 16 s [6–50]
Moridani et al. [27]	Cardiac	HRV	ECG	R-peak detection by Pan and Tompkins’ algorithm. *Time domain analysis*: SDNN, RMSSD, NN50, pNN50. *Frequency domain analysis*: LF, HF, VLF, LF/HF. *Poincaré plot analysis*: SD1, SD2, SD2/SD1	Retrospective	Focal epilepsy (7)	11/±6 h	NA	NA	Sens: (overall: 88.3%) PPV: NA FAR: NA DL: NA
Pavei et al. [28]	Cardiac	HRV	ECG	*Noise filtering*: Visual artifact inspection, high- and low-pass Butterworth filters. QRS detection algorithm by Kohler. *Time domain analysis*: SDNN, RMSSD. *Frequency domain analysis*: LF, HF (using FFT). *SampEn*: Entropy changes. Lorenz plot analysis: CSI, CVI	Retrospective	Temporal lobe epilepsy (12)	34/171 h	FOIA (34)	34.5 SD 7.5	Sens: (overall: 94.1%) PPV: (overall: 95.6%) FAR: (overall: 0.49/h) DL: NA
Qaraqe et al. [29]	Cardiac	HRV	Single-lead ECG	*Noise filtering*: Baseline estimation and denoising with sparsity. QRS detection algorithm. Outlier removal, linear interpolation. *Time–frequency analysis*: MP-WVD algorithm. SVM classifier for EEG features	Retrospective	Focal epilepsy (7)	68/NA	FOA, FOIA, FOBTC	43.6 [26–65]	ECG: Sens: 96.4% [75–100%] PPV: NA FAR: 5.4/h [1.5–9.5/h] DL: 13.1 s [8–20.5] ECG + EEG: Sens: 100% PPV: NA FAR: 1.6/h [0–3.5/h] DL: 12.3 s [3–26]
Vandecasteele et al. [30]	Cardiac	HRV/PRV	180° eMotion Faros and Empatica E4 smart-watch	R-peak detection by Pan–Tompkins’ algorithm. HRV analysis: Method of Varon. PRV analysis: Method of Lázaro. Seizure detection algorithm with SVM classifier by de Cooman. *Feature extraction*: HR_peak_, HR_base_, and STDHR_base_	Retrospective	Temporal lobe epilepsy (11)	47/701 h	NA	42.7 [19-67]	Wearable ECG: Sens: 64% (overall: 70%) PPV: 2.03% (overall: 2.15%) FAR: 2.35/h (overall: 2.11/h) DL: NA Hospital ECG: Sens: 57% (overall: 57%) PPV: 2.22% (overall: 1.93%) FAR: 2.05/h (overall: 1.92/h) DL:NA PPG: Sens: 33% (overall:32%) PPV: 1.43% (overall: 1.12%) FAR: 1.88/h (overall:1.80/h) DL: NA
Varon et al. [43]	Cardiac	QRS morphology	Single-lead ECG	R-peak detection via Pan–Tomkins’ algorithm. *Algorithm 1*: principal component analysis for changes in QRS morphology. *Algorithm 2*: ictal acceleration of HR quantified by using phase-rectified signal averaging (PRSA)	Retrospective	1) Children with refractory epilepsy (37) 2) Women with epilepsy (5)	1) 98 2) 10/±5 h^c^	1) FOS (48) (28 frontal, 20 temporal) GOS (50) (29 T/TC 11 MC, 10 absences) 2) FOS (10)	1) 9.2 [3–16] 2) [31–48]	Algorithm 1^d^: Sens: 89.5% (F1), 86% (G1), 100% (F2) PPV: 85.7% (F1), 57.3% (G1), 52.6% (F2) FAR: NA DL: NA Algorithm 2: Sens: 100%(F1), 90%(G1), 100%(F2) PPV: 90.5% (F1), 77.5% (G1), 71.4% (F2) FAR: NA DL: NA
Elmpt, van et al. [32]	Cardiac	HR	2-lead ECG	R identification (increase signal > 250 µV in 10 ms). *HR*: baseline (60 s before increase), periictal period (120 s after), SD, min and max. * Detection threshold*: +2SD from baseline for ≥ 5QRS complexes. Curve-fitting algorithm and onset detection algorithm	Retrospective	Severe epilepsy (10)	104/9 h	T, TC, MC, atypical absences	34.1 [21–50]	Sens: NA PPV: NA FAR: NA DL: NA HR increase in 48.1% of seizures. Great variability in sens and PPV. Better performance when combined with ACC
Osorio et al. [34]	Cardiac	HR	ECG	True beat range determined (30–180 bpm). * 5 s moving window*: RRI determination. *Time of beat sequence (TOBS) *: RHR, 4 threshold values (T) and 3 duration values (D)	Retrospective	Focal onset epilepsy (81) Dataset 1 (41), 2 (40)	241/6935 h	FOS	NA	Lowest settings T, D Sens: 98.8% PPV: NA FAR: 9.5/h (1), 7.2/h (2) DL: NA Highest settings T, D Sens: 85.5% PPV: NA FAR: 1.1/h (1), 0.7/h (2) DL: NA
Boon et al. [31]	Cardiac	HR	VNS—AspireSR	Relative HR increase > 1 s above threshold (≥ 20%, ≥ 40%, ≥ 60% above baseline HR)	Prospective	Refractory epilepsy (16)	66/NA	FOS (8), FOA (26), FOIA (31), FOBTC (17), U (5)^e^	39.6 SD 13.4 [19–66]	Threshold > 20%: Sens: 16/27 = 59.3% PPV: NA FAR: 7.2/h [95% CI 5.31–9.94] DL: 6.0 s [− 112–105] Threshold > 40%: Sens: 8/23 = 34.8% PPV: NA FAR: 2.7/h [95% CI 1.70–3.91] DL: 27.5 s [0–57] Threshold > 60%: Sens: 3/16 = 18.8% PPV: NA FAR: 0.5/h [95% CI 0.20–0.96] DL: 35.0 s [4–40]
Hampel et al. [33]	Cardiac	HR	VNS—AspireSR	Heartbeat sensitivity threshold 50% compared to baseline	Prospective	Refractory epilepsy (1)	12/68 h	FOS with hyperkinetic movements	29	Sens: 92% PPV: 8% FAR: 1.88/h (*n* = 128) DL: 7.4 s (± 5)
Andel, van et al. [36]	Combined	HR, ACC	Shimmer sensor (chest ECG + 3D ACC)	*Algorithm 1*: No. of s in which summed waveform length > fixed threshold within a fixed window. Detection if no. > window length/4. * Algorithm 2*: HR > threshold. * Algorithm 3*: combination of summed waveform length OR HR	Retrospective	Epilepsy (43)	86/402 h^f^	Major motor (86) (18 TC, 41 T, 18 HM, 9 Cluster)	Median 15 [2–65]	All seizures: Sens: 60% (A1), 56% (A2), 71% (A3) PPV: NA FAR: 0.5/h (A1), 0.3/h (A2), 0.7/h (A3). DL: NA Clinically urgent seizures: Sens: 74% (A1), 71% (A2), 87% (A3) PPV: NA FAR: 0.6/h (A1), 0.3/h (A2), 0.8 (A3). DL: NA
Cogan et al. [2]	Combined	HR, SpO2, EDA	Affectiva Q-curve and Nonin WristOx2 sensor	Seizure pattern analysis (HR↑, SpO2↓, EDA↑). Biosignal algorithm. Personalized parameters (*P*)	Retrospective	Focal epilepsy (10)	26/340 h	FOIA (23), FOBTC (2), GTCS (1)	41.8 [21–64]	3 Sensors (*n* = 6): Sens: 100%, 100% (P) PPV: 86%, 100% (P) FAR: 0.015/h, 0.000/h (P) DL: NA
Goldenholz et al. [37]	Combined	HR, QTc, SpO2	Single-lead ECG, Radical-7	*SpO2*: ictal drop, provided it remained > 50%. Optimal balance 80–86%. Calculation of HR and QTc by Bazette method	Retrospective	Refractory epilepsy (45)	151/7104 h	FOS (119), FOBTC (32)	40 [14–68]	Sens^g^: (overall: 81–94% (FOBTC), 25–36%(FOS)) PPV: NA FAR: (overall: 0.4–2.4/h) DL: NA
Heldberg et al. [38]	Combined	EDA, ACC	Empathica E3 wristband	*EDA*: low-pass filter, cutoff frequency of 1.5 Hz. * Time windows*: 10 s 50% overlap and 5 min 80% overlap. Decomposing signal (Ledalab algorithm). Feature extraction (56) of EDA and ACC. kNN (11 features) and random forest (26 features) classifiers	Retrospective	Epilepsy (8)	55/540 h	Motor seizures (21), nonmotor (34)	NA	kNN classifier: Sens: 76.2% (M), 97.1% (non-M) PPV: 4.6% (M), 9.7% (non-M) FAR: NA DL: NA Random forest: Sens: 90.5% (M), 85.3% (non-M) PPV: 5.6% (M), 12.3% (non-M) FAR: NA DL: NA
Onorati et al. [39]	Combined	EDA, ACC	Empatica E3, E4, iCALM	10 s sliding epochs (75% overlap), feature extraction, classifier, decision thresholds. (3 sets: Poh’s (19), larger (46), reduced (25))	Retrospective	Epilepsy (69) (24 children, 45 adults)	55/5928 h	FOBTC (49), FOTC (6)	Median 14/37 [4–60]	Sens: 83.6% (C1), 92.7% (C2), 94.6 (C3) PPV: 39% (C1), 50% (C2), 51% (C3) FAR: 0.29/day (C1), 0.21 (C2), 0.20 (C3) DL: 31.2 s (C1), 29.3 s (C2), 29.3 s (C3)
Poh et al. [40]	Combined	EDA, ACC	Custom-built wrist-worn biosensors	10 s epochs, sliding window with 75% overlap, preprocessing, 19 time, frequency, and nonlinear features extracted to form feature vectors. SVM to classify vectors as (non)seizure. Cross-validation	Retrospective	Focal epilepsy (7)	16/688 h^h^	FOBTC (16)	10 SD 4.6	Nonpatient-specific: Sens: 14/16 = 88% PPV: NA FAR: 0.04/h (*n* = 28) DL: NA Semi-patient specific: Sens: 15/16 = 94% PPV: NA FAR: 0.04/h DL: NA

*ACC* accelerometry, *CSI* cardiac sympathetic index (SD2/SD1), *CVI* cardiac vagal index (log_10_(SD2xSD1)), *DL* detection latency, *ECG* electrocardiogram, *EDA* electrodermal activity*, EEG* electroencephalography, *FAR rate* false alarm rate*, FFT* fast-Fourier transformation*, FN* false negative, *FOA* focal onset aware seizures*, FOBTC* focal onset to bilateral tonic–clonic, *FOIA* focal onset with impaired awareness*, FOS* focal onset seizures*, FOTC* focal onset tonic–clonic*, GOS* general onset seizures*, GTC* generalized tonic–clonic, *HF* high frequency (0.15–0.4 Hz)*, HR* heart rate*, HR*_*base*_ average HR over the 60 s before the start of the HR increase, *HR*-*diff* heart rate differentiation, *HR*_*peak*_ peak HR at the end of the HR increase, *HRV* heart rate variability*, IHR* instantaneous heart rate (inverse of RRI)*, LF* low frequency (0.04–0.15 Hz), *LO(P)O*-*CT* leave-one-(patient)-out crosstesting, *mCSI* modified CSI (SD2^2/SD1), *MC* myoclonic*, MP*-*WVD algorithm* matching pursuit and Wigner–Ville distribution algorithm, *NA* not applicable*, No.* number, *NPS* non-patient-specific*, PPG* photoplethysmography, *PRV* pulse rate variability, *RHR* relative heart rate, *RRI* R–R interval*, RMSSD root* mean square of difference in adjacent RRIs, *SampEn* sample entropy*, s* seconds, *SD* standard deviation*, Sens* sensitivity, *SPS* semi-patient-specific, *STDHR*_*base*_ standard deviation of the HR over the 60 s before the start of the HR increase, *SVM* support vector machine*, TP* total power, *TRT* total recording time*, T/TC* tonic or tonic–clonic*, U* unknown, *VHF* very high frequency (0.4–0.5 Hz), *VLF* very low frequency (0.0001–0.04 Hz), *VNS* vagus nerve stimulation*, y* years

^a^Data used for validation

^b^Lorenz plot = Poincaré plot

^c^Data used for validation

^d^*F1* focal seizures in children, *G1* generalized seizures in children (F1 + G1 = training set), *F2* focal seizures adult, used for validation

^e^Specified for all seizures, only 66 analyzed

^f^Training and test data combined

^g^Percentage of evaluable data

^h^3525 h without seizures were also tested for false positives

Most studies had small sample sizes (median population size 14, IQR 7–26). The number of seizures analyzed per patient tended to be low (median number of seizures per participant 3, IQR 2–7). The total recording time used to validate the algorithm varied from 7 min to 158 h per person (median recording time per participant 34 h, IQR 3–86 h), but was not specified in two studies. Seizure onset was mostly focal (*n* = 14) [8, 22, 24–26, 28, 30, 31, 33, 34, 37, 39, 40, 42], but was focal and generalized in some (*n* = 4) [2, 23, 35, 42] or not specified in others (*n* = 3) [32, 36, 38].

All four performance measures (sensitivity, PPV, FAR, and DL) were only reported in three out of 21 studies [22, 33, 39]; eight studies reported three [2, 23–25, 28, 30, 31, 42], eight studies reported two [8, 26, 34, 36–38, 40, 43], one study reported one [41], and one study only reported sensitivity and PPV data for some of the subjects [32].

### Heart rate analysis

Heart rate was monitored using single or multiple lead electrocardiography (ECG) in 14 of 18 studies [8, 22–26, 28, 32, 34–37, 42, 43]. Alternative methods included photoplethysmography (PPG) in a wearable sensor (*n* = 2) [2, 30] and an implanted heart rate sensor (AspireSR) (*n* = 2) [31, 33].

Heart rate measurement was done using various methods of R-peak detection, including those proposed by Pan and Tompkins [30, 41], Kohler [28], Yeh and Wang [22–24], or unspecified methods [8, 25, 26, 31–34, 42]. Some studies applied noise filtering techniques to diminish false R-peak detection, including high- and low-pass noise filters [8, 22–24, 26, 30] or a specific algorithm (baseline estimation and denoising with sparsity) [42].

One case study prospectively assessed a HR algorithm using a vagal nerve stimulation (VNS) device with a fixed HR sensitivity threshold [33]. Alarms were generated when the HR augmentation exceeded 50% of the baseline HR. Eleven out of 12 seizures were detected (sensitivity 92%), together with 128 false alarms (FAR 1.88/h; 68 h recordings). A second prospective validation study of the same VNS device compared different HR thresholds (≥ 20%, ≥ 40%, and ≥ 60% increases from baseline) in 16 adults with refractory epilepsy [31]. Lower thresholds resulted in higher sensitivity and higher FAR than higher thresholds (e.g., sensitivity 59.3% and FAR 7.2/h for threshold ≥ 20% vs. sensitivity 18.8% and FAR 0.5/h for thresholds ≥  60%).

Similar effects of varying the thresholds (for both the relative HR increase and the duration of HR increase) were reported in two studies on retrospectively validated HR algorithms [32, 34]. A follow-up using the same dataset examined different factors that may influence the probability of seizure detection [44]. The best regression model was created with variables including age, gender, etiology, seizure class, and years with epilepsy.

### Heart rate variability (HRV)

All of the HRV-focused studies performed retrospective validations [8, 22–26, 28, 30, 41, 42]. Different HRV features were selected and specific feature thresholds were classified as ‘ictal’ or ‘interictal.’ Nine out of ten HRV studies applied linear analysis [8, 22–25, 28, 30, 41, 42] using time domain [22–25, 28, 30, 41, 42] and frequency domain [8, 25, 28, 41, 42] features. Time domain analysis focuses on the instantaneous HR; the interval between two normal QRS complexes, abbreviated to ‘NN.’ Different time domain features, such as the mean NN interval or the distribution of NN have been used for seizure detection. Four studies extracted and classified these time domain features using a support vector machine (SVM) classifier and validated the same HRV algorithm in different populations [22–24, 30]. The first retrospective study of 17 people with temporal lobe epilepsy found a mean sensitivity of 83.2% with a FAR of 2.01/h [22]. The second study extracted ECG or PPG data from three different heart rate sensors worn by 11 adults with temporal lobe epilepsy [30]. The best performance was obtained using a wearable ECG device, with a sensitivity of 64% and a FAR of 2.35/h. A third study tested the algorithm in 28 children and showed a higher overall sensitivity (81.3%) and a lower FAR (0.75/h) [23]. Performance, particularly FAR, improved when applying a patient-specific heuristic classifier. The latter was confirmed in the fourth study of data from 19 people with temporal lobe epilepsy from a pre-existing epilepsy database [24]. The authors also proposed an adaptive seizure detection algorithm, and showed that similar results were obtained with simulated ‘real-time’ user feedback.

Frequency domain analysis is used to extract the frequency components of the HR signal, each with its own physiological footprint: low frequency (LF 0.04–0.15 Hz), high frequency (HF 0.15–0.40 Hz), very low frequency (VLF 0.0001–0.04 Hz), and very high frequency (VHF 0.4–0.5 Hz). Different frequencies were identified by power spectral density analysis of HRV in four studies [8, 25, 28, 41], and two studies sped up this process by applying an efficiency algorithm [fast Fourier transform (FFT)] [8, 28]. The LF/HF ratio , reflecting the balance of sympathetic and parasympathetic function, was examined in two studies [25, 41]. One of these studies tested a seizure detection algorithm combining both time and frequency domain features on 11 focal seizures upon awakening [25]. Ten of the 11 seizures were detected prior to seizure onset (sensitivity 91%, DL − 494 ± 262 s). Another study of seven adults with focal epilepsy that used time–frequency analysis of HRV based on a combination of the matching-pursuit and Wigner–Ville distribution algorithms reported a sensitivity of 96.4% with high FAR (5.4/h) [42]. Combining ECG and EEG algorithms yielded better performance (sensitivity 100%, FAR 1.6/h).

To assess the dynamic properties of ictal HR changes, nonlinear analysis can be applied, such as a Lorenz (or Poincaré) plot. This method plots the current R–R interval against the next R–R value. Standard deviations in the transverse (SD1) and longitudinal (SD2) directions of these plots can be calculated, and higher ratios of SD2/SD1 reflect increased sympathetic tone. These ratios can be used in seizure detection algorithms, since an increase in sympathetic tone is often seen during the preictal and early ictal phases. One small retrospective study proposed the modified cardio sympathetic index (mCSI) as a new measure in seizure detection that reflects the sympathetic tone [26]. A seizure detection algorithm based on changes in mCSI yielded a sensitivity of 88% in five people with temporal lobe epilepsy (FAR not reported). A larger follow-up study of adults with focal epilepsy compared frequency domain analysis with Lorenz plot analysis [8]. mCSI appeared more sensitive, but FARs were not reported.

The two remaining studies of HRV combined linear and nonlinear analysis [28, 41]. The first retrospective study of seven people with focal epilepsy reported an overall sensitivity of 88.3% with a specificity of 86.2% after selecting an optimal performance threshold for each patient [41]. The second study combined time–frequency and Lorenz plot analysis with a second nonlinear analysis of ‘sample entropy’ [28]. This parameter quantifies the regularity and complexity of a time series, and entropy decreases can be seen during the ictal phase. Applying all of these methods together to ECG data from twelve temporal lobe epilepsy patients resulted in overall sensitivity of 94.1% with a FAR of 0.49/h.

Another retrospective study reported two different seizure detection algorithms based on changes in QRS morphology (algorithm 1) and cardiorespiratory interactions (algorithm 2) [35]. The first algorithm captured five consecutive QRS complexes, aligned them with respect to the R peak, and assembled them into one QRS matrix. Principal component analysis was used to select different features from this QRS matrix. This process was repeated for every heart beat, which resulted in a sensitivity of 89.5–100% for detecting focal onset seizures and 86% for generalized onset seizures. The second algorithm was based on the well-known modulatory effects of respiration on HRV. These cardiorespiratory changes were quantified using phase-rectified signal averaging—a methodology used to detect quasi-periodicities in nonstationary signals such as the resampled RR interval time series—and were used for seizure detection. Slightly better performance was achieved by the second algorithm, which yielded a sensitivity of 100% for focal onset seizures and 90% for generalized onset seizures. In this study, 10.4–90% of the generated alarms were false, and this percentage was lower for the second algorithm.

### Combining autonomic parameters

All multimodal autonomic algorithms were retrospectively validated. A combination of three biosignals, measured by two different devices, was used for seizure detection in a study of ten subjects with focal epilepsy [2]. An algorithm based on a specific seizure pattern of increased HR, decreased SpO2, and increased EDA was able to detect all seizures in six out of ten patients with a low FAR of 0.015/h. Specific thresholds of HR, QTC, and SpO2 were combined in an algorithm tested on a larger study population of 45 people with refractory epilepsy [37]. Only half of the collected data was used for analysis, and a sensitivity of 81–94% was found for focal to bilateral tonic–clonic seizures, while focal seizures without bilateral spreading showed worse performance, with a sensitivity of 25–36%. Overall FAR ranged from 0.4–2.4/h.

Three other retrospective validation studies combined EDA and accelerometry (ACC), measured with one device [28–40]. Different classifiers were used to select features of EDA and ACC. The first study tested two machine learning algorithms, the k-nearest neighbor (kNN) and random forest classifiers. The kNN classifier achieved the best results with 11 features, and was most sensitive for nonmotor seizures (sensitivity 97.1%, FAR not reported). The random forest classifier selected 26 features and showed its best performance with motor seizures (sensitivity 90.5%, FAR not reported). A second study used a SVM classifier to extract 19 features (16 ACC and 3 EDA) [40]. Fourteen out of 16 focal onset seizures with bilateral spreading were detected (sensitivity 88%) and FAR was 0.04/h. The same feature set was used in the third study and compared to a larger (40 ACC and 6 EDA) and a reduced (22 ACC and 3 EDA) feature set [39]. Retrospectively tested on 24 children and 45 adults with focal epilepsy, the reduced set showed the best performance (sensitivity 94.6%, FAR 0.20/day).

A multicenter study combined HR and ACC measures in 95 people with nocturnal major motor seizures [36]. Data from only 23 patients could be used to retrospectively validate three different algorithms based on changes in HR, ACC, and ‘HR or ACC.’ Clinically urgent seizures were detected well (sensitivity 71–87%), but FAR was relatively high (2.3–6.3/night), with wide variation between subjects.

### Quality of the included studies

According to the QUADAS-2 criteria, the overall quality of the included studies was medium–high (Table 2). Seventeen out of 21 studies were at risk of bias, mainly due to an undefined patient selection process and fitting of the algorithm [2, 8, 22–26, 30, 32, 34, 37–43]. There was concern regarding the applicability of the selected patients in three studies, because the populations consisted of children only and/or were not well described [23, 25, 33]. Concerns about the applicability of the index test (i.e., the tested algorithm) arose in nine studies, mainly because the algorithm was fitted to one dataset [2, 8, 23, 25, 28, 30, 32, 36, 37].

**Table 2:**
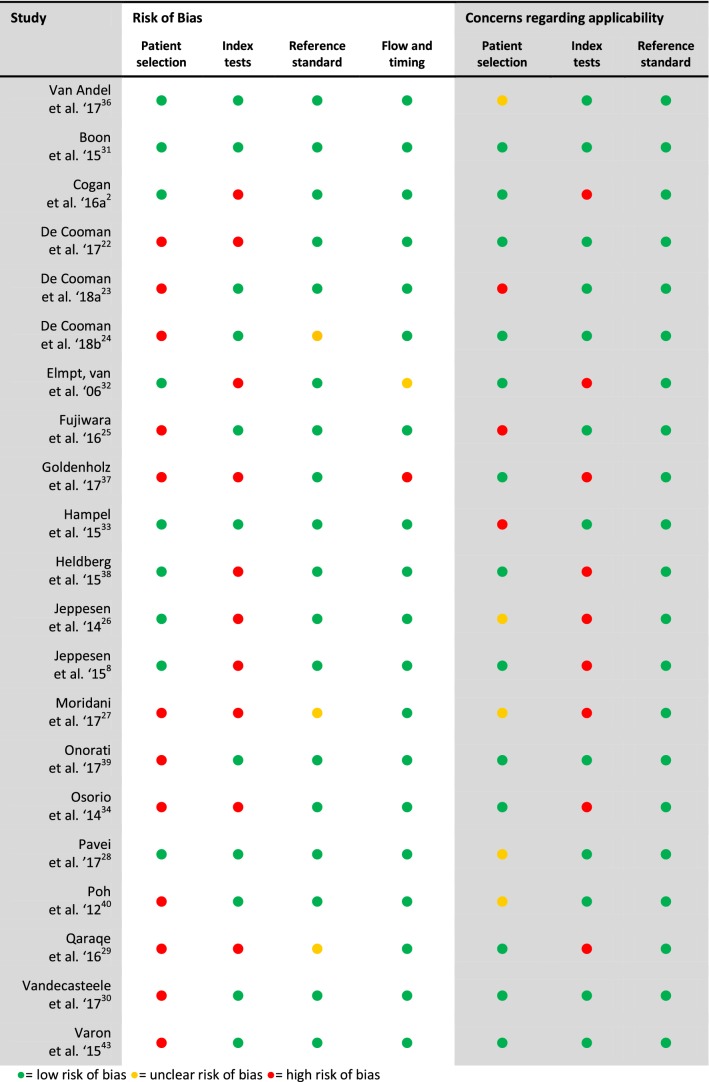
Quality of the included studies according to QUADAS-2

Based on the standards for the clinical validation of SDDs proposed by Beniczky and Ryvlin [21], most studies were classified as phase 1 proof-of-principle studies, whereas three were classified as phase 0 initial studies [34, 41, 42], and only one as a phase 2 study on a dedicated SDD [31] (Table 3). Seven other studies also tested a dedicated device but included small population sizes or did not address the safety of the device and were therefore classified as phase 1 [2, 30, 33, 36, 38–40]. Ten studies trained and tested their algorithm on the same dataset [2, 8, 22, 26, 32, 34, 37, 40–42], and only four used a predefined algorithm or cutoff values [30, 31, 33, 36]. Eighteen studies used video-EEG as reference standard; the remaining three used EEG or ECoG without video recordings [34, 41, 42].

**Table 3:** Quality of validation studies of seizure detection, as assessed using standards proposed by Beniczky and Ryvlin

Study	Subjects			Recordings					Analysis and alarms				Reference standard		Study phase
	Simulation/healthy subjects	No. of people with seizures	No. of seizures	Conventional methods	Dedicated device	Continuous	Multicenter	Offline/retrospective	Training and testing using the dataset	Predefined algorithm and cutoff values	Real time	Blinded	Video or video-EEG recordings	Information from pt and caregivers	
Van Andel et al. [36]	–	20–50	≥ 75	–	+	+	+	+	–	+	–	–	+	–	1
Boon et al. [31]	–	10–20	30–75	–	+	+	+	–	–	+	+	–	+	+	2
Cogan et al. [2]	–	10–20	15–30	–	+^a^	+	–	+	+	–	–	–	+	–	1
De Cooman et al. [22]	–	10–20	≥ 75	+	–	+	–	+	+	–	–	–	+	–	1
De Cooman et al. [23]	–	20–50	≥ 75	+	–	+	+	+	–	–	–	–	+	–	1
De Cooman et al. [24]	–	10–20	≥ 75	+	–	+	–	+	–	–	–	–	–^b^	–^c^	1
van Elmpt et al. [32]	–	10–20	≥ 75	+	–	+	–	+	+	–	–	–	+	–	1
Fujiwara et al. [25]	–	10–20	1–15	+	–	–	+	+	–	–	–	–	+	–	1
Goldenholz et al. [37]	–	20–50	≥ 75	+	–	+	–	+	+	–	–	–	+	–	1
Hampel et al. [33]	–	≥ 1	1–15	–	+	+	–	–	–	+	+	–	+	+	1
Heldberg et al. [38]	–	1–10	30–75	–	+	+	–	+	–	–	–	–	+	–	1
Jeppesen et al. [26]	–	1–10	1–15	+	–	–	–	+	+	–	–	–	+	–	1
Jeppesen et al. [8]	–	10–20	30–75	+	–	–	–	+	+	–	–	–	+	–	1
Moridani et al. [27]	–	1–10	1–15	+	–	+	–	+	+	–	–	–	–^b^	–	0
Onorati et al. [39]	–	≥ 50	30–75	–	+	+	+	+	–	–	–	–	+	–	1
Osorio et al. [34]	–	≥ 50	≥ 75	+	–	+	+	+	+	–	–	–	–^d^	–	0
Pavei et al. [28]	–	10–20	30–75	+	–	–	–	+	–	–	–	–	+	–	1
Poh et al. [40]	–	1–10	15–30	–	+	–	–	+	+	–	–	–	+	–	1
Qaraqe et al. [29]	–	1–10	30–75	+	–	–	–	+	+	–	–	–	–^b^	–	0
Vandecasteele et al. [30]	–	10–20	30–75	+	+	+	–	+	–	+	–	–	+	–	1
Varon et al. [43]	–	20–50	≥ 75	+	–	–	–	+	–	–	–	–	+	–	1

Phase 0: initial studies performed when initiating or developing a novel method. Phase 1: proof-of-principle studies. Phase 2: studies of a dedicated seizure detection device. Phase 3: studies allowing the final confirmation of safety and accuracy. Phase 4: in-field studies of seizure detection devices in the home environments of the patients, addressing aspects related to usability

*No.* number, *pt* patient

^a^Two different devices combined

^b^Available database with EEG recordings

^c^Simulated real-time feedback on detections

^d^ECoG without video

## Discussion

The overall quality of studies on seizure detection using autonomic parameters is low. Small population sizes, short follow-up periods, and high study heterogeneity raise concerns about the applicability of the results. Available studies are mainly initial or proof-of-principle studies that lack long-term and real-time ambulatory monitoring, which is needed to obtain more reliable performance data and usability outcomes.

HR- or HRV-based algorithms are most frequently applied, but it is hard to compare the results of different studies due to wide variation in the detection techniques used and a lack of FAR data (Table 4). Additionally, FAR, when mentioned, is high for these studies and exceeds acceptable limits for daily practice. We could not compare the performance of HR- and HRV-based algorithms due to the wide variety of study designs employed. HRV-based algorithms seem attractive given their short detection latency, but they still require prospective validation. HRV is, however, situation dependent and affected by exercise, stress, respiration, and sleep stage [45–47]. These confounding factors make it more challenging to distinguish ictal patterns from non-ictal ones, resulting in lower accuracy [48]. Also, similar activation of the autonomic nervous system can occur before physiological arousal or other sleep-related movements [49].

**Table 4:** Performance of seizure detection algorithms grouped according to dataset size

Study	Validation of algorithm				Performance of algorithm			
	No. of subjects	No. of seizures/TRT	Type of seizures	Algorithm	Sensitivity (%)	FAR	PPV (%)	DL (s) [range]
Large datasets								
van Andel et al. [36]	23	86/402 h^a^	All major motor^b^	Heart rate	60	0.5/h	NA	NA
				Movement	56	0.3/h	NA	NA
				Hart rate or movement	71	0.7/h	NA	NA
		59	Clinically urgent seizures^c^	Heart rate	74	0.6/h	NA	NA
				Movement	71	0.3/h	NA	NA
				Hart rate or movement	87	0.8/h	NA	NA
De Cooman et al. [22]	17	127/918 h	FOS, including TCs		83.2 [50–100]	2.01/h [0.88–3.52/h]	7.9% [0.4–21%]	13.3[− 18.2–54.3]
De Cooman et al. [23]	28	107/695 h	Convulsive and clinical subtle seizures	Patient-independent	Overall: 81.3	Overall: 0.75/h	NA	NA
				Patient-specific	Overall: 77.6	Overall: 0.33/h	Overall: 30.7	19.1
De Cooman et al. [24]	19	153/2833 h	FOS, including TCs (only clinical seizures)	Patient-independent	Overall: 78.4	Overall: 1.73/h	Overall: 2.4	NA
				Patient-specific	Overall: 76.5	Overall: 1.09/h	Overall: 3.7	NA
				Adaptive	Overall: 77.1	Overall: 1.24/h	Overall: 3.3	NA
Goldenholz et al. [37]	45	151/7104 h	FOS, including TCs		Overall: 81–94 (FOBTC) 25–36 (FOS)^e^	Overall: 0.4–2.4/h	NA	NA
Onorati et al. [39]	69	55/5928 h	FOS, all TCs	Classifier 1	83.6	0.29/day	39	31.2
				Classifier 2	92.7	0.21/day	50	29.3
				Classifier 3	94.6	0.20/day	51	29.3
Medium datasets								
Boon et al. [31]	16	66/NA	Different types of FOS, including TCs	Threshold > 20%	59.3	7.2/h [95% CI 5.31–9.94]	NA	6.0 [-112–105]
				Threshold > 40%	34.8	2.7/h [95% CI 1.70–3.91]	NA	27.5 [0–57]
				Threshold > 60%	18.8	0.5/h [95% CI 0.20–0.96]	NA	35.0 [4–40]
Heldberg et al. [38]	8	55/540 h	Motor (M) and non-M seizures	kNN classifier	76.2 (M) 97.1 (non-M)	NA	4.6 (M) 9.7 (non-M)	NA
				Random forest	90.5 (M) 85.3 (non-M)	NA	5.6 (M) 12.3(non-M)	NA
Jeppesen et al. [8]	17	47/ ± 27 h	FOS, including TCs		81: (mCSI-100) (overall: 74, mCSI-100)	NA	NA	16 [6–50]
Osorio et al. [34]	81	241/6935 h	FOS	Lowest settings T,D Datasets (1) and (2)	98.8	9.5/h (1) 7.2/h (2)	NA	NA
				Highest settings T,D Datasets (1) and (2)	85.5	1.1/h (1) 0.7/h (2)	NA	NA
Pavei et al. [28]	12	34/171 h	FOIA		Overall: 94.1	Overall: 0.49/h	Overall: 95.6	NA
Poh et al. [40]	7	16/688 h^f^	FOS, all TCs	Non-patient-specific	88	0.04/h (*n* = 28)	NA	NA
				Semi-patient-specific	94	0.04/h	NA	NA
Qaraqe et al. [29]	7	68/NA	FOS, including TCs	ECG	96.4 [75–100]	5.4/h [1.5–9.5/h]	NA	13.1 [8–20.5]
				ECG + EEG	100	1.6/h [0–3.5/h]	NA	12.3 [3–26]
Vandecasteele et al. [30]	11	47/701 h	FOIA	Wearable ECG	64 (overall: 70)	2.35/h (overall: 2.11/h)	2.03 (overall: 2.15)	NA
				Hospital ECG	57 (overall: 57)	2.05/h (overall: 1.92/h)	2.22 (overall: 1.93)	NA
				PPG	33 (overall:32)	1.88/h (overall:1.80/h)	1.43 (overall: 1.12)	NA
Small datasets								
Cogan et al. [2]	6	10/340 h	FOIA and TCs	3 Sensors	100	0.015/h	86	NA
				Personalized	100	0.000/h	100	NA
Elmpt, van et [32]	10	104/9 h	Motor seizures (T, TC, MC) and atypical absences		NA^d^	NA	NA	NA
Fujiwara et al. [25]	14	11/69 h	FOS (awake)	*T*^2^ statistics	Overall: 55	Overall: 1.2/h	NA	− 524 ± 216
				*Q* statistics	Overall: 91	Overall: 0.7/h	NA	− 494 ± 262
Hampel et al. [33]	1	12/68 h	FOS with hyperkinetic movements		92	1.88/h	8	7.4 (± 5)
Jeppesen et al. [26]	5	11/13 h	FOIA		88 (CSI-30) (overall: 73, CSI-30, mCSI-50)	NA	NA	− 5–60
Moridani et al. [27]	7	11/± 6 h	FOS		Overall: 88.3	NA	NA	NA
Varon et al. [43]	42	108/± 5 h	FOS and GOS, including T, TC, MC, and absences	Algorithm 1^g^	89.5 (F1)	NA	85.7 (F1)	NA
					86 (G1)		57.3 (G1)	
					100 (F2)		52.6 (F2)	
				Algorithm 2^f^	100 (F1)	NA	90.5 (F1)	NA
					90 (G1)		77.5 (G1)	
					100 (F2)		71.4 (F2)	

*CSI* cardiac sympathetic index, *DL* detection latency, *ECG* electrocardiogram, *EEG* electroencephalography, *FAR rate* false alarm rate, *FOBTC* focal onset to bilateral tonic–clonic, *FOIA* focal onset with impaired awareness, *FOS* focal onset seizures, *h* hour, *MC* myoclonic, *mCSI* modified cardiac sympathetic index, *NA* not applicable, *No*. number, *PPG* photoplethysmography, *s* seconds, *T* tonic, *TCs* tonic–clonic seizures, *TRT* total recording time

^a^Training and test set combined

^b^Including tonic–clonic, tonic, hypermotor, and cluster (series of at least five tonic or myoclonic spasms within 3 min)

^c^When attendance or intervention was deemed necessary, based on seizure severity, postictal arousal state, breathing difficulties, and distress

^d^High variability in sensitivity and PPV

^e^Percentage of evaluable data

^f^3525 h without seizures were also tested for false positives

^g^*F1* focal seizures in children, *G1* generalized seizures in children (F1 + G1 = training set), *F2* focal seizures in adults, used for validation

Multimodal algorithms might help to lower FARs. A retrospective study of seven children with tonic–clonic seizures validated different unimodal and multimodal algorithms on the same dataset. All combinations of multimodal sensors, including ECG, EMG, and ACC, showed at least 75% lower FAR [50]. Studies differentiating outcome according to seizure type showed diverse results, indicating that that different seizure types may require different detection techniques. Multimodal techniques can provide a solution to this problem [51]. Another solution could be personalizing or tailoring the algorithm. One study group studied two different personalization strategies and calculated the number of seizures required for accurate tailoring [52]. The authors proposed an initialization phase to tailor an existing predefined algorithm to a patient-specific algorithm. Six to eight seizures seemed sufficient to set individual thresholds [52]. Another retrospective multicenter study proposed an automatic adaptive HRV algorithm and tested it on a database of 107 nocturnal seizures from 28 children [23]. After an initialization phase of five seizures, the personalized algorithm resulted in lower FARs compared to those obtained with the patient-independent algorithm. A follow-up study proposed an adaptive classifier with real-time user feedback that presented similar performance; this method might be better accepted in daily practice [24].

## Conclusion

Autonomic function alterations seem to represent an attractive tool for timely seizure detection. Unimodal autonomic algorithms cannot, however, reach acceptable performance: while most algorithms are quite sensitive, false alarm rates are still too high. Multimodal algorithms and personalization of the algorithm are important strategies to improve performance. Larger, prospective, home-based studies with long-term follow-up are needed to validate these methods and to demonstrate the added value of SDDs in clinical care.

## Electronic supplementary material

Below is the link to the electronic supplementary material.

Supplementary material 1 (DOCX 15 kb)
