# Supplementary material for: Ictal autonomic changes as a tool for seizure detection: a systematic review
Source: Clin Auton Res. 2018 Oct 30;29(2):161–81. doi: 10.1007/s10286-018-0568-1 (PMC6459795; doi:10.1007/s10286-018-0568-1)
Supplement: Supplementary file 1 — Supplementary material 1 (DOCX 15 kb) [file 10286_2018_568_MOESM1_ESM.docx]

| **Search PubMed on May 22^nd^ 2018** |
| --- |
| ("Heart Rate"[Mesh] OR heart rate[tw] OR heart rates[tw] OR heart rate monitoring[tw] OR heart rate monitor[tw] OR heart rate monitors[tw] OR heart rate variability[tw] OR heart rate response[tw] OR heart rate responses[tw] OR pulse[tw] OR pulse wave[tw] OR pulse waves[tw] OR pulse rate[tw] OR pulse rates[tw] OR pulse rate monitoring[tw] OR pulse rate monitor[tw] OR pulse rate monitors[tw] OR pulse monitoring[tw] OR pulse monitor[tw] OR pulse monitors[tw] OR photoplethysmography[tw] OR cardiac chronotropism[tw] OR chronotropy[tw] OR chronotropic[tw] OR heartbeat[tw] OR heartbeats[tw] OR heartbeat monitoring[tw] OR heartbeat monitor[tw] OR heartbeat monitors[tw] OR heart beat[tw] OR heart beats[tw] OR heart beat monitoring[tw] OR heart beat monitor[tw] OR heart beat monitors[tw] OR pulsation[tw] OR "Autonomic Nervous System"[Mesh] OR Autonomic Nervous System[tw] OR vegetative Nervous System[tw] OR autonomic system[tw] OR autonomic function[tw] OR autonomic symptoms[tw] OR autonomic symptom[tw] OR "Sweat"[Mesh] OR Sweat[tw] OR transpiration[tw] OR transpire[tw] OR sweating[tw] OR sweating rate[tw] OR electrodermal activity[tw] OR electrodermal response[tw] OR saturation[tw] OR oxygenation[tw]) AND ("Epilepsy"[Mesh] OR epilep*[tw] OR epileptic seizure[tw] OR epileptic seizures[tw] OR convulsion[tw] OR convulsions[tw] OR seizure[tw] OR seizures[tw])AND ("Monitoring, Ambulatory"[Mesh] OR "Signal Processing, Computer-Assisted"[Mesh] OR "Monitoring, Physiologic"[Mesh] OR detecting[tw] OR seizure detection[tw] OR seizure monitoring[tw] OR seizure observation[tw] OR seizure detection device[tw] OR seizure detection devices[tw] OR algorithm[tw] OR algorithms[tw]) |
| **Search Embase on May 22^nd^ 2018** |
| ('heart rate'/exp OR 'heart rate' OR 'pulse rate'/exp OR 'pulse rate' OR 'heart rate':ti,ab OR 'heart rates':ti,ab OR 'heart rate monitoring':ti,ab OR 'heart rate monitor':ti,ab OR 'heart rate monitors':ti,ab OR 'heart rate variability':ti,ab OR 'heart rate response':ti,ab OR 'heart rate responses':ti,ab OR 'pulse':ti,ab OR 'pulse wave':ti,ab OR 'pulse waves':ti,ab OR 'pulse rate':ti,ab OR 'pulse rates':ti,ab OR 'pulse rate monitoring':ti,ab OR 'pulse rate monitor':ti,ab OR 'pulse rate monitors':ti,ab OR 'pulse monitoring':ti,ab OR 'pulse monitor':ti,ab OR 'pulse monitors':ti,ab OR ‘photoplethysmography’:ti,ab OR 'cardiac chronotropism':ti,ab OR 'chronotropy':ti,ab OR 'chronotropic':ti,ab OR 'heartbeat':ti,ab OR 'heartbeats':ti,ab OR 'heartbeat monitoring':ti,ab OR 'heartbeat monitor':ti,ab OR 'heartbeat monitors':ti,ab OR 'heart beat':ti,ab OR 'heart beats':ti,ab OR 'heart beat monitoring':ti,ab OR 'heart beat monitor':ti,ab OR 'heart beat monitors':ti,ab OR 'autonomic nervous system'/exp OR 'autonomic nervous system' OR 'sweating'/exp OR 'sweating' OR 'autonomic nervous system':ti,ab OR 'vegetative nervous system':ti,ab OR 'autonomic system':ti,ab OR 'autonomic function':ti,ab OR 'autonomic symptoms':ti,ab OR 'autonomic symptom':ti,ab OR 'sweat':ti,ab OR 'transpiration':ti,ab OR 'transpire':ti,ab OR 'sweating':ti,ab OR 'sweating rate':ti,ab OR ‘electrodermal activity’:ti,ab OR ‘electrodermal response’:ti,ab OR ‘saturation’:ti,ab OR ‘oxygenation’:ti,ab) AND ('seizure'/exp OR 'seizure' OR 'epileptic seizure':ti,ab OR 'epileptic seizures':ti,ab OR 'convulsion':ti,ab OR 'convulsions':ti,ab OR 'seizure':ti,ab OR 'seizures':ti,ab) AND ('ambulatory monitoring'/exp OR 'ambulatory monitoring' OR 'physiologic monitoring'/exp OR 'physiologic monitoring' OR 'signal processing'/exp OR 'signal processing' OR 'seizure detection':ti,ab OR ‘detecting’:ti,ab OR 'seizure monitoring':ti,ab OR 'seizure observation':ti,ab OR 'seizure detection device':ti,ab OR 'seizure detection devices':ti,ab OR ‘algorithm’:ti,ab OR ‘algorithms’:ti,ab) AND [embase]/lim NOT [medline]/lim |

*Supplementary table 1: search strategy*
